# Lightweight Thermal Compensation Technique for MEMS Capacitive Accelerometer Oriented to Quasi-Static Measurements

**DOI:** 10.3390/s21093117

**Published:** 2021-04-30

**Authors:** Javier Martínez, David Asiain, José Ramón Beltrán

**Affiliations:** 1 Department of Electronic Engineering, Escuela Universitaria Politécnica de la Almunia, C/Mayor 5, La Almunia de Doña Godina, 50100 Zaragoza, Spain; dasiain@unizar.es; 2 Department of Electronic Engineering and Communications, Universidad de Zaragoza, C/María de Luna 1, 50018 Zaragoza, Spain; jrbelbla@unizar.es

**Keywords:** MEMS, accelerometer, thermal drift, thermal compensation, tilt measurements

## Abstract

The application of MEMS capacitive accelerometers is limited by its thermal dependence, and each accelerometer must be individually calibrated to improve its performance. In this work, a light calibration method based on theoretical studies is proposed to obtain two characteristic parameters of the sensor’s operation: the temperature drift of bias and the temperature drift of scale factor. This method requires less data to obtain the characteristic parameters, allowing a faster calibration. Furthermore, using an equation with fewer parameters reduces the computational cost of compensation. After studying six accelerometers, model LIS3DSH, their characteristic parameters are obtained in a temperature range between 15 °C and 55 °C. It is observed that the Temperature Drift of Bias (TDB) is the parameter with the greatest influence on thermal drift, reaching 1.3 mg/°C. The Temperature Drift of Scale Factor (TDSF) is always negative and ranges between 0 and −400 ppm/°C. With these parameters, the thermal drifts are compensated in tests with 20 °C of thermal variation. An average improvement of 47% was observed. In the axes where the thermal drift was greater than 1 mg/°C, the improvement was greater than 80%. Other sensor behaviors have also been analyzed, such as temporal drift (up to 1 mg/h for three hours) and self-heating (2–3 °C in the first hours with the corresponding drift). Thermal compensation has been found to reduce the effect of the latter in the first hours after power-up of the sensor by 43%.

## 1. Introduction

Micro-electromechanical systems (MEMS) [1] have multiple advantages over traditional technology. Their small size, low cost, and low power consumption have promoted their use in smartphones. And the expansion of smartphones and tablets has contributed greatly to the improvement of MEMS technology and sensors. The MEMS accelerometer of smartphones can be used in multiple applications, such as user transportation means detection [2], pedestrian recognition [3], or structural integrity monitoring [4].

Outside the smart-phone field, accelerometers are widely used in monitoring applications. They have been applied to railway infrastructure [5], geotechnical monitoring [6], geostructural safety [7], surface failure of slopes [8], bridge structural monitoring [9], and even tree property measurements [10].

However, one major disadvantage of MEMS capacitive accelerometers is their temperature dependence. This effect, although small compared to some other technologies, reduces the viability of MEMS accelerometers in medium to high precision applications where thermal variations are expected. Thermal stresses can temporarily or permanently alter the behavior of the units [11]. These alterations are not deterministic; different units of the same model exposed to the same processes exhibit different thermal sensitivities [12].

Multiple ways have been proposed to improve the thermal behavior of MEMS accelerometers. Some studies propose alternative design techniques of the internal structures [13,14,15,16,17]. Other studies reduce the thermal drift by compensating its effects on the resonant circuits [18], implementing sub-circuits [19], implementing application specific integrated circuit [20], taking advantage of the capacitor parasitic resistance [21], or isolating them in a micro oven-control system with controlled temperature [22]. These techniques are used to improve the technology capabilities, but, to improve the performance of an accelerometer in application, other techniques are required.

Some software techniques have been proposed to obtain thermal compensation parameters for the accelerometers [23,24]. These techniques are based on a polynomial equation in which the parameters are obtained with regression methods. To ensure the accuracy of the parameters, the tests must be performed for each working temperature and inclination. Alternative methods consists of using neural networks to predict and compensate the thermal behavior [25]. In both methods, the calibration process for each DUT (Device Under Test) is time consuming and hard to compute, increasing with each degree of the polynomial model or with each neuron added. Generally, external hardware and software must be used to achieve calibration. This can increase significantly the cost, as the calibration must be performed individually.

Many modern MEMS accelerometers and IMUs integrate a temperature sensor. Some of the more advance series, like the ADIS series by Analog Devices, also include the compensation techniques and are individually factory calibrated. This greatly improves its performance, but also the cost.

The objective of this paper is to research a lightweight and fast method to achieve the thermal characterization and compensation of MEMS accelerometers, and test its performance. This work proposes a straightforward model according to theoretical analysis of MEMS capacitive accelerometers [26]. The proposed model contains only two characteristic parameters for every axis. Both parameters are directly related to the accelerometers thermal behavior: the Temperature Drift of Bias (TDB) and the Temperature Drift of Scale Factor (TDSF). This method does not require a polynomial regression, reducing the amount of data and time required to obtain the compensation parameters.

## 2. The MEMS Accelerometer

### 2.1. Working Principle

**Figure 1:**
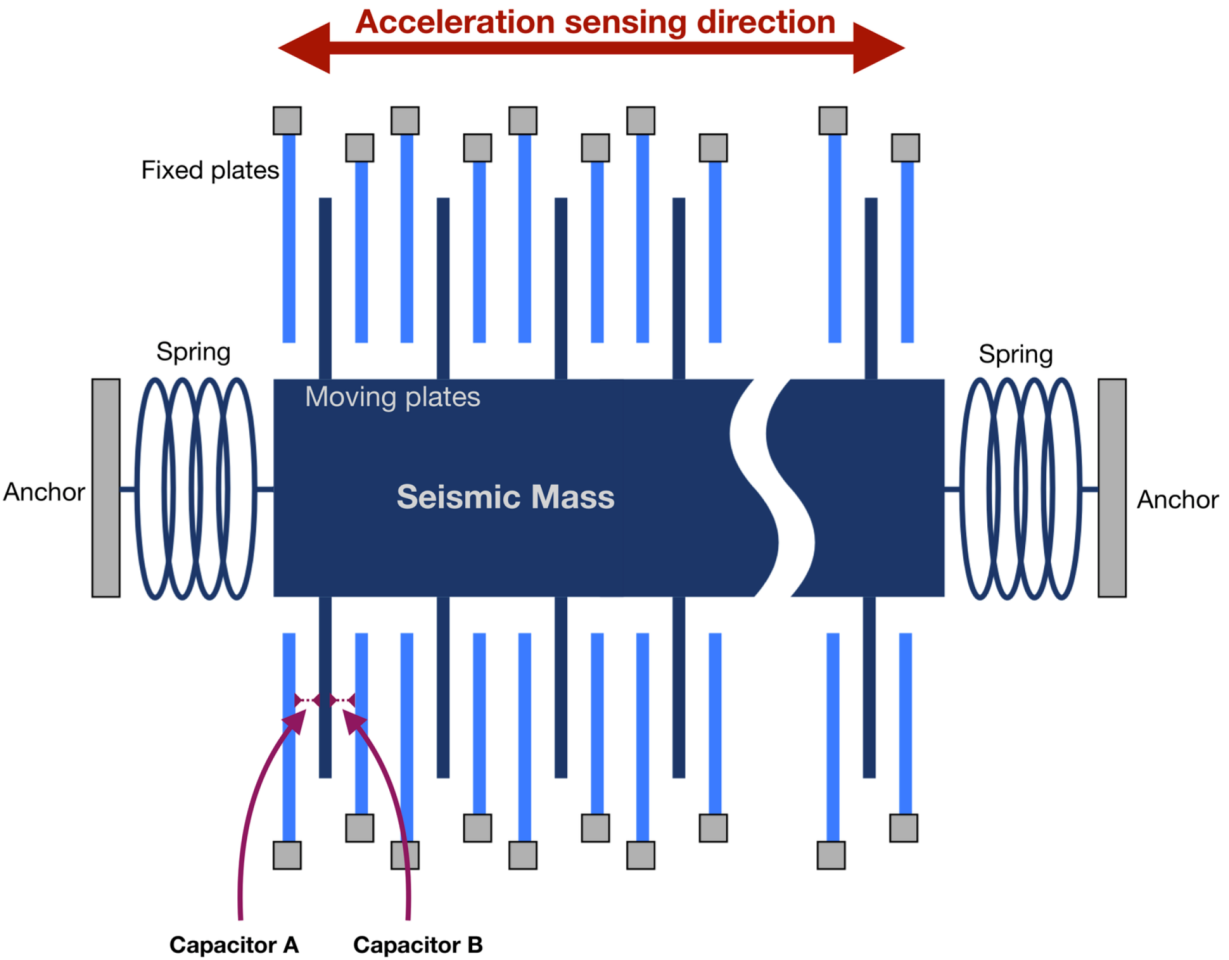
Working principle of a capacitive accelerometer.

Capacitive accelerometers rely on a change in electrical capacitance in response to acceleration. They utilize the properties of opposed plate capacitors. One plate of each capacitor is fixed, and the other is attached to a seismic mass between springs [27]. Multiple pairs of capacitors are used with the same seismic mass, as shown in Figure 1. Any external acceleration displaces the seismic mass, modifying the distance between the capacitors plates, and, therefore, their capacitance. The difference between the values of opposing capacitors varies proportionally to the applied acceleration. Both capacitances can be measured with voltage pulses [11].

The micromechanical structure is made of silicon. Silicon is a temperature-sensitive material and its physical characteristics vary greatly with temperature. Specific structural designs can reduce this temperature dependence [17]. Two single-axis structures can be arranged perpendicular to each other on the same plane creating a biaxial MEMS accelerometer. To allow for the third sensitive axis, another sensing technique has to be used. There are multiple ways to allow this perpendicular axis sensitivity, for example, the torsional springs technique [27]. This way a triaxial accelerometer can be fabricated in one plane. However, this third axis usually suffers from lower sensitivity, higher thermal drifts and, in general, worse performance.

MEMS capacitive accelerometers are very sensitive to soldering processes. Thermal stresses and dilatations can induce permanent mechanical stresses in the interior of the MEMS. Externally, uneven cooling of the solder tin can also induce mechanical stresses. Both of these effects permanently affect the Zero-g level of the sensor. Therefore, it is necessary to calibrate the devices after the soldering process.

### 2.2. Triaxial Accelerometer Calibration

The design of a triaxial accelerometer consists of three orthogonally arranged single axis sensors. Manufacturing inaccuracies cause misalignment between the sensitive axes and the accelerometers body. Furthermore, the sensitive axes are not orthogonal and cross sensitivities appear. Compensating for these effects is especially important to achieve reliable tilt measurements. It is also necessary to calibrate the individual sensitivity and bias for each axis. The output of a triaxial accelerometer can be represented as Equation (1).

(1) $$\left[\begin{array}{c} X_{0}\\ Y_{0}\\ Z_{0} \end{array}\right]=\left[\begin{array}{ccc} S_{X} & 0 & 0\\ 0 & S_{Y} & 0\\ 0 & 0 & S_{Z} \end{array}\right]\cdot \left[\begin{array}{ccc} A_{XX} & A_{YX} & A_{ZX}\\ A_{XY} & A_{YY} & A_{ZY}\\ A_{XZ} & A_{YZ} & A_{ZZ} \end{array}\right]\cdot \left[\begin{array}{c} X\\ Y\\ Z \end{array}\right]+\left[\begin{array}{c} b_{X}\\ b_{Y}\\ b_{Z} \end{array}\right],$$

where $X_{0}$, $Y_{0}$, and $Z_{0}$ are the accelerometer measurements; *X*, *Y*, and *Z* are the ideal accelerations on the orthogonal axis; $S_{X}$, $S_{Y}$, and $S_{Z}$ are the scale factor of the accelerometer axes; $b_{X}$, $b_{Y}$, and $b_{Z}$ are the bias of the axes; and $A_{XX}$, $A_{YX}$, $A_{ZX}$, $A_{XY}$, $A_{YY}$, $A_{ZY}$, $A_{XZ}$, $A_{YZ}$, and $A_{ZZ}$ are the transformation parameters that relate the orientation of the sensitive axes to the orthogonal axes of the body [28].

Multiple algorithms can be used to obtain the calibration parameters [29,30]. A simple method consists of using the six positions where an axis is perpendicular to the earth plane. After obtaining all the data, the factors are obtained with the least square method.

Temperature affects each sensitive axis individually, so they must be analyzed separately, without mutually affecting each other due to mechanical calibration. Once the thermal drift is compensated, the misalignment, bias and sensitivity calibration will be performed to obtain reliable measurements of inclination.

### 2.3. Tilt Measurement Techniques for Accelerometers

**Figure 2:**
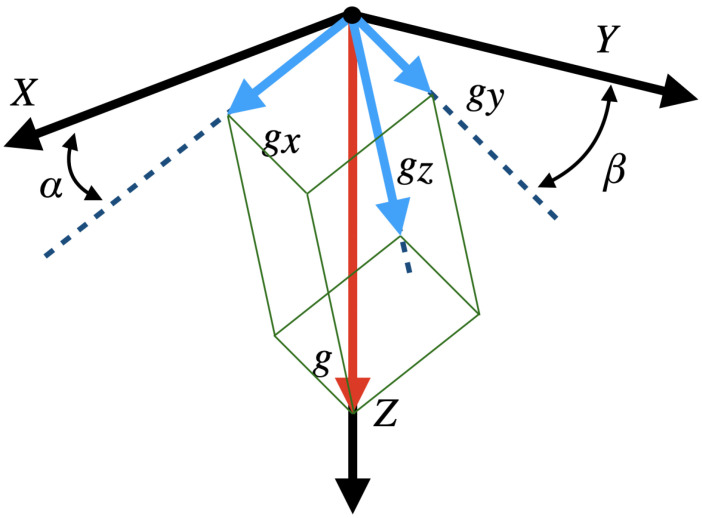
Axes accelerations and Euler angles.

Measurement of tilt using accelerometers takes advantage of the constant gravity vector. Each axis measurement ($g_{x}$, $g_{y}$, and $g_{z}$) represents the projection of the gravity vector on that axis (see Figure 2) [31]. With them, it is possible to determine the Euler pitch, α, and roll, β, angles. Depending on the number of axes and mathematical formula used to compute the angles, the measurement range, sensor sensitivity, and alignment dependence may change [32].

With three orthogonal accelerations, it is possible to compute the Euler angles in different ways. The performance of some of them is greatly affected by the orientation of the sensor [32]. The sensitivity of tilt measurements drops down to zero near ±90°. Therefore, to obtain the best performance in the maximum range, all three axes have to be taken into account.

The two Euler angles α and β can also be computed if only the acceleration in two axes is available. However, if one of the axis used is close to ±1 g, the accuracy will be reduced. Therefore, the best solution would be to mount both sensitive axes parallel to the ground. This allows the use of Equations (2) and (3) to obtain the Euler angles [33].

(2) $$\alpha =\arcsin\frac{g_{x}}{g},$$

(3) $$\beta =\arcsin\frac{g_{y}}{g}.$$

These equations could be used assuming that g is exactly 1000 mg, or computing it as the geometric sum of $g_{x}$, $g_{y}$, and $g_{z}$.

### 2.4. Thermal Behavior

Capacitive MEMS accelerometers are sensitive to temperature variations. These variations are produced by both external and internal phenomena; for example, internal electronics produce heat due to ohmic loss. Temperature changes affect the internal structure in multiple ways: changes in the Young’s modulus, thermal deformations and thermally induced stresses [34]. Thermal drifts are linked to variations in the stiffness of the beams and springs of the structure, caused by manufacturing imperfections [35].

Other effects, such as imperfections in the soldering process or the different coefficients of thermal expansion between the glass substrate and the silicon structure, can also induce mechanical stresses when subjected to temperature variations.

In working conditions, the thermal behavior of the MEMS accelerometer is characterized by the Temperature Drift of Bias and the Temperature Drift of Scale Factor [17,26,36]. TDB is expressed in mg/°C, it can greatly affect the output value depending on the temperature, and it is directly proportional to the beam stiffness differences. Since manufacturing imperfections are random, so are the sign and value of the TDB. This randomness requires a specific thermal calibration for each DUT to ensure the best performance.

The TDSF generates a change in the sensitivity of the sensor as a function of temperature. This generates a drift proportional to both the temperature and the raw acceleration of the axis. The TDSF value is expressed in ppm/°C and is always negative [26], which implies that the sensitivity of the sensor always decreases when the temperature increases.

The data provided by manufacturers shows a similar behavior. In Reference [11], typical “Sensitivity change versus temperature” (TDSF) and “Zero-g level change versus temperature” (TDB) data are provided. By using a reference temperature $T_{R}$, which the manufacturer sets at 25 °C, the output value of a MEMS accelerometer, *X*, can be expressed as the real value, $X_{0}$, plus the bias error and the scale factor error, as shown in Equation (4), where *T* represents the DUTs’ temperature.

(4) $$X\left(T\right)=X_{0}+\left(T-T_{R}\right)\left(TDB+TDSF\cdot X_{0}\right).$$

#### Thermal Compensation

To ensure the best possible performance of the accelerometer, the thermal drift has to be compensated. The most common method is to fit a linear polynomial to the MEMS behavior. Niu et al. [24] proposed a third order polynomial equation (Equation (5)) to achieve the calibration. This equation does not take into account the change in sensitivity with the temperature, since the raw acceleration is not present in the equation. All the parameters of the polynomial models correspond to different orders of TDB. The dispersion between the A1 parameter of the three axes in both studied accelerometers agrees with the randomness of sign and value previously described. The result of this equation is not the compensated value, but the thermal induced error (*E*) according to the temperature (*T*). It has to be added, or subtracted, to the measurements to get the compensated acceleration value.

(5) $$E\left(T\right)=A_{0}+A_{1}\cdot T+A_{2}\cdot T^{2}+A_{3}\cdot T^{3}.$$

To account for variations in the sensors sensitivity, the raw acceleration has to be included in the thermal compensation technique. This results in a compensation surface with more parameters. Ruzza et al. [23] proposed a second order surface (defined by Equation (6)) to obtain the thermal induced error (*E*) from the raw acceleration (*X*) and temperature (*T*).

(6) $$E\left(T,X\right)=p_{00}+p_{10}T+p_{01}X+p_{20}T^{2}+p_{11}T\cdot X+p_{02}X^{2}.$$

They generate different surfaces for cooling and heating cycles. This can compensate for the possible hysteresis of the system, but increases the complexity of the technique, since two surfaces are used for each axis, and the temperature gradient must be taken into account before compensation. The two surfaces are not coincident, and it can lead to sudden changes in the output value.

Each coefficient in Equation (6) can be related with a specific characteristic of the system, and compared with the theoretical Equation (4):

- $p_{00}$ = Bias in LSBs at 0 °C.
- $p_{10}$ = Data variation proportional to temperature in LSBs/°C. Same as TDB.
- $p_{01}$ = Sensibility of the acceleration in LSBs. Same as $S_{X}$ in Equation (1).
- $p_{20}$ = Second order TDB. It does not have much effect, since it is three orders lower than $p_{10}$.
- $p_{11}$ = Sensitivity change because of temperature. Same as TDSF.
- $p_{02}$ = Second order non-linearity sensibility in LSBs. Typical order of $10^{-8}$.

This technique can lead directly to the compensated acceleration value, $X_{0}$. For that to happen, $p_{01}$ must be close to 1, adding the raw acceleration to the output value.

Ruzza et al. also detected that the thermal drifts in a small population of sensors is random [12]. In all these studies, the *Z* axis shows a different behavior compared to the *X* and *Y* axes. This may be due to the different sensitive axis design mentioned in Section 2.1.

The randomness and magnitude of the thermal drifts requires an individual calibration for each accelerometer. This process can be expensive and time consuming, as each accelerometer has to be analyzed at all temperatures and inclinations, and then the model parameters have to be extracted.

We propose a fast compensation technique based on Equation (4). By using the two most relevant parameters (TDB and TDSF), the computational cost and time required for each DUT can be reduced. Each parameter, if not properly adjusted, can lead to errors. Therefore, fewer compensation parameters can mean a more robust compensation technique, as there are fewer potential sources of error.

This thermal compensation does not have to give the exact acceleration measurement; the objective is to obtain a value that does not depend on temperature. After this, a mechanical calibration is carried out that allows us to obtain reliable measurements of acceleration and inclination.

## 3. Methodology

To study the characterization of a MEMS capacitive accelerometer according to Equation (4), it is necessary to analyze its response to temperature variations in multiple orientations. This data can be used to obtain the TDB and TDSF parameters for each DUT and compensate for its thermal drifts. Six accelerometers will be studied simultaneously; in this way, the dispersion between DUTs can be perceived.

### 3.1. The Accelerometer—Device under Test

For this paper, we will use the LIS3DSHTR MEMS accelerometer manufactured by STMicroelectronics [11]. This accelerometer has been chosen for being a low cost model of a company with significant importance in the inertial MEMS market. In addition, its small size and low energy consumption favor its use in IoT systems or in portable devices.

The LIS3DSH is a capacitive three-axis linear accelerometer that has dynamically selectable full scales of ±2 g, ±4 g, ±6 g, ±8 g, and ±16 g. This device also comes with one embedded temperature sensor with an 8 bit resolution. It is factory calibrated to give an output of 1 LSB per °C.

Communication with the LIS3DSH can be done through I2C and SPI serial interfaces. Other typical characteristics of this MEMS accelerometer are shown in Table 1.

Some of these characteristics, such as bias and TDB, can vary depending on multiple factors (PCB mounting, thermal stresses, mechanical stresses). Therefore, the manufacturer does not guarantee typical specifications.

This accelerometer has a built-in self-test. This allows the user to unbalance the internal capacitive bridge and simulate an acceleration. In the *Z* axis, this simulated force is four times bigger than on the *X* and *Y* axes. This is an indication that the *Z* axis has four times lower sensitivity and requires more amplification. It is probably caused by the different mechanical design of the sensitive axis.

In many tilt measurement applications, and especially in monitoring applications, acceleration is not expected to experience rapid variations. Consequently, the accelerometer sampling rate and the antialiasing filter frequency can be set to low values. The working range will not exceed 1g, allowing to achieve a better resolution. The configuration loaded into the DUTs is shown in Table 2. All other configuration registers are not modified.

### 3.2. Hardware—TestBench

**Figure 3:**
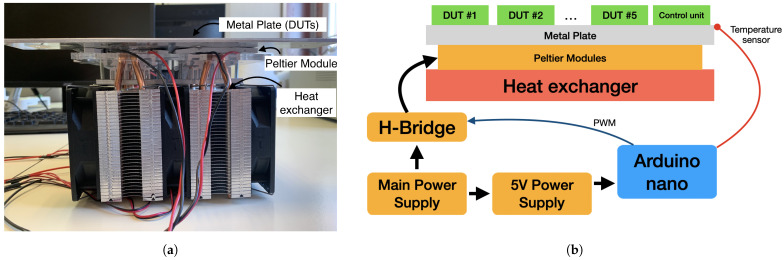
Testbench used to induce the temperature variations during the tests. (**a**) Peltier modules with the metal plate and the heat exchanger. (**b**) Working diagram.

All the DUTs will be studied simultaneously to ensure that the temperature stresses are similar across all of them. In this way, the results become easier to compare. All the DUTs are in contact with the same metal plate. The temperature of this plate is changed using four Peltier modules distributed throughout the area. The modules allow to generate stable temperatures from 10 °C to 65 °C. Lower temperatures could be reached, but water might condensate on the pcbs. Temperatures above 65 °C are not recommended to avoid system degradation. Power is delivered to the Peltier modules through an H-Bridge for a better temperature control. The H-Bridge is controlled with an Arduino Nano Board, which has a temperature sensor on the metal plate for a close loop control (see Figure 3).

**Figure 4:**
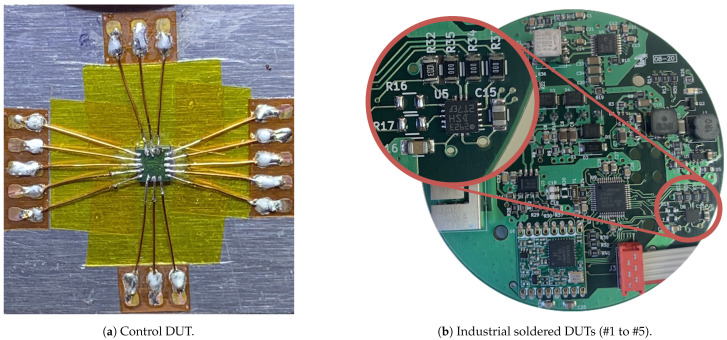
DUTs used during tests.

Each DUT includes one LIS3DSH capacitive MEMS Accelerometer and a SAMD21G18 MCU. Five of the DUTs are attached to an identical printed circuit board and have been produced with the same industrial soldering processes, undergoing the same thermal and mechanical stresses (see Figure 4b). The last DUT will be the control DUT, which has not undergone any industrial soldering process. The pads of the control DUT have been carefully soldered to minimize the thermal stress on the package (see Figure 4a). Soldering wire with a melting point of 179 °C and a soldering station with temperature control has been used. The DUTs’ temperature was always controlled to ensure that the internal structures suffered no thermal stresses.

### 3.3. Tests Conditions

Before starting the tests with temperature variations, the DUTs are studied in a fixed position and at room temperature for 20 h. This is used to get a cool start and a reference behavior. Some characteristics, such as signal noise, self-heating, or temporary drifts, can be analyzed. The main tests, which will be used for TDB and TDSF, are performed only after the accelerations are stable and no temporary drifts are detected.

**Figure 5:**
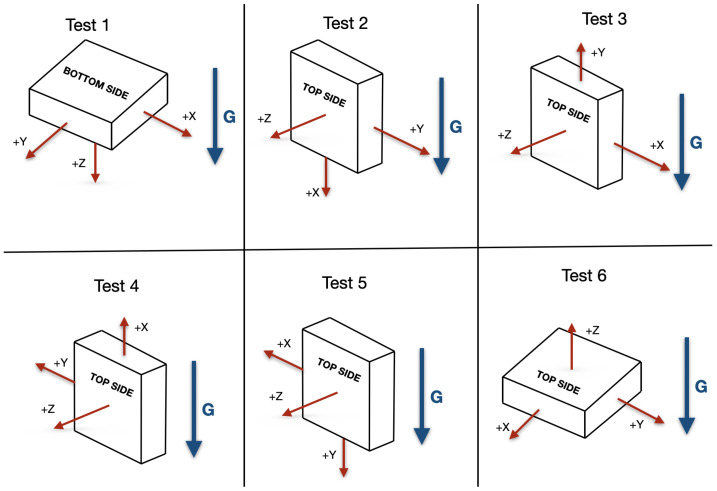
Orientations during the six calibration tests.

The main tests are carried out in six different orientations, shown in Figure 5. It is not necessary for the orientations to be set very precisely, as the actual acceleration value used to subsequently calculate the TDSF will be taken during each test as the acceleration at 25 °C. The TDB only depends on variations, so it is not affected by the actual value of acceleration. These are the minimum number of tests that provide information about the full application range (±1 g) for all three axes.

The temperature profiles during the tests follow the Soak method [24]. This method is based on generating stable temperatures during the tests. Three different temperatures are used during each test. The fast temperature variations can help analyze the response time of the sensor and other effects, such as hysteresis [37]. The time spent in each temperature step must ensure that the entire transient response has been mitigated, and it is set at two hours for each step. Therefore, a full cycle will take eight hours to complete. Three uninterrupted cycles will be logged for each orientation, with a total duration of 24 h for each orientation.

The tests require only three temperature points to allow characterization. This allows the tests to be performed without specialized equipment, such as Peltier modules or thermal chambers. Only one heat source and one cooling source are required. These test conditions are chosen to allow thermal calibration without expensive equipment.

After the six main tests are completed, a verification test is performed. In this case, the temperature profile is triangular with a period of 12 h and an amplitude of 20 °C (from 25 °C to 45 °C). This test is carried out to simulate thermal variations closer to working conditions and to test the compensation performance. It is carried out for three days without interruption.

### 3.4. Signal Processing

The DUTs data are captured at 3.125 Hz, containing information about capture time, *X* axis raw acceleration (in LSBs), *Y* axis raw acceleration (in LSBs), *Z* axis raw acceleration (in LSBs), and temperature (in °C). The temperature is sampled with the LIS3DSH integrated temperature sensor. For a better comprehension of the results and comparison with other works, the acceleration is transformed to be expressed in mg.

**Figure 6:**
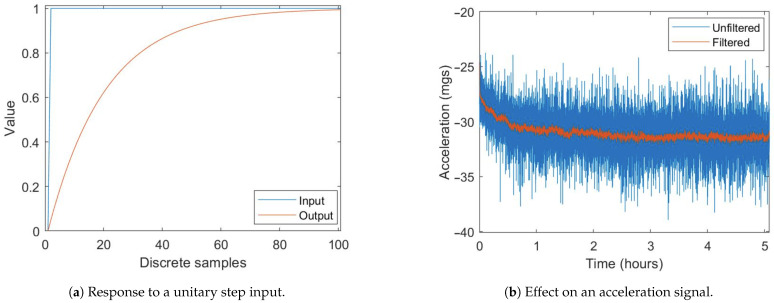
Exponential filter behavior.

The captured data has a large white noise component. Therefore, an exponential filter is used to reduce it (Equation (7)). This filter is chosen over others because of its low computing load. This allows it to be implemented in applications in low cost MCUs or CPUs. Since the data are quasi-static the filtering factor, α, can be set at a low value, increasing the response time. The filtering factor is set at 0.05; therefore, the response time of the filter is 43 samples, equivalent to 13.76 *s* at 3.125 Hz. Its effect on the signal can be appreciated in Figure 6.

(7) $$y_{n}=\left(1-\alpha \right)y_{n-1}+\alpha \cdot x_{n}.$$

## 4. Results

The graphical representation of the captured data shows many characteristics that need to be further analyzed, such as noise, temperature drifts, temporary drifts, and self-heating. All DUTs, including the control DUT, exhibit similar behavior and characteristics, but to different extents.

### 4.1. Thermal Drift

**Figure 7:**
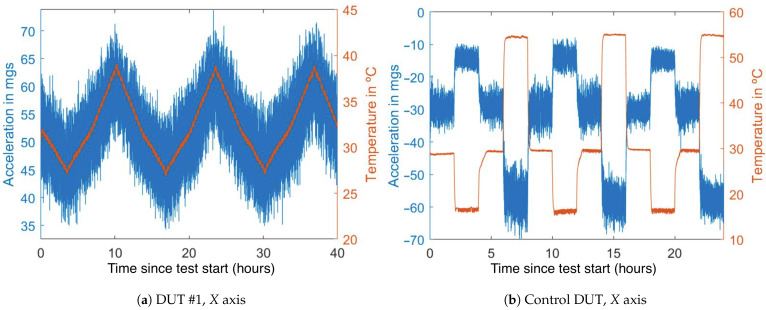
Simultaneous temperature (in red) and acceleration (in blue) variations during the tests.

It is clear that the temperature changes induce large variations in the measured acceleration (see Figure 7). If this effect is left uncompensated, it can lead to large errors. The first test shows that it can be higher than 1 mg/°C. This means that thermal variations of 10 °C can cause more error than temporary drift or noise, so it is a characteristic that must be compensated for to improve the performance of the sensors. This is especially important in long-term tilt measurement applications, as the temperature changes can lead to errors that can not be compensated for with standard filters.

The temperature variations induce immediate acceleration variations, there are no significant delays or response time between both variations. Furthermore, the drifts seem to be proportional to the thermal variations, and no significant hysteresis effect appears.

### 4.2. Self-Heating

**Figure 8:**
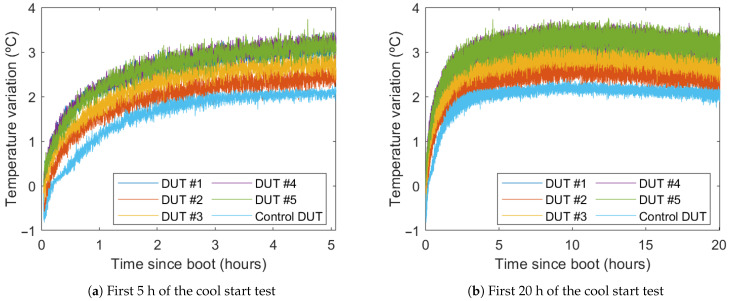
Temperature variation during the cool start test.

During the first moments of the start-up test, the temperatures of all DUTs rise (see Figure 8). This effect is caused by the heat produced in both the external and internal electronic circuits, due to ohmic losses. The temperature increase is up to 2 °C in the control DUT and between 2.5 °C and 3.5 °C in the PCBs (Printed Circuit Boards) on which the DUTs have been placed. This difference is caused by two factors: more heat produced in the PCB DUTs due to the nearby electronics (MCU and power supply), and better heat dissipation of the control DUT, as it is attached directly to a metal plate rather than soldered to a printed circuit board.

The thermal increase is concentrated in the first two or three hours after cool start. After that point, the temperature rises less than 1°C in ten hours. After the ten-hour mark, the temperature can be considered stable. (The temperature falling at the end of the test is caused by the cooler room temperature during the night.)

This initial thermal variation will cause a thermal drift that could be compensated for to improve the system performance during the first hours. Drift produced by this self-heating should be similar to the thermal drift produced by a similar variation in external temperature. Therefore, thermal compensation should also reduce the drift caused by this initial self-heating.

### 4.3. Temporal Drift

**Figure 9:**
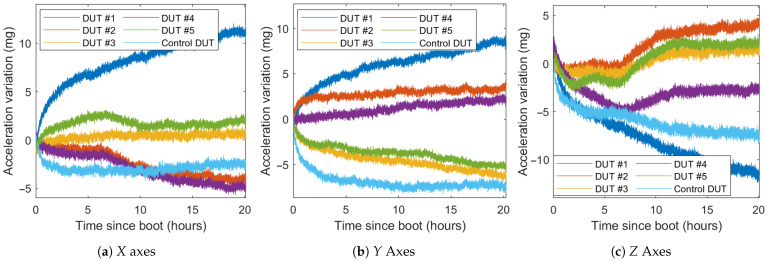
Acceleration value of the DUTs during the cool start test.

During the constant temperature test, the acceleration values of the axes do not remain stable (see Figure 9). The drift during the first hours of the test can be caused by the self-heating effect. However, the remaining drifts after the first hours can not be attributed to thermal drifts because the temperature does not change significantly.

DUTs #2, #4, and #5 modify their behavior in the *Z* axis between the fifth and tenth hour of the test. This variation does not appear in DUTS #1 or #3. In general, this adds another unreliability factor to the *Z* axis of the sensor. This effect is also present, although much lower, in the *X* axis in the same DUTs. The DUTs average and worst temporary drifts for multiple time segments are shown in Table 3.

This drift is unique for each axis and DUT, although they have similar behaviors. The drift is very high in the first two hours of the test, up to 3 mg/h. As self-heating becomes less dominant, the drift progressively reduces, and the average drift after the first ten hours is around 0.13 mg/h.

The control DUT has a high drift in the first hour, significantly higher than the average drift. After three hours, this drift is considerably reduced, becoming lower than 0.1 mg/h after ten hours. This may be due to the better thermal conductivity of the control DUT.

The maximum difference error caused by the drift in 20 h is up to 11 mg for the *X* axis, 9 mg for the *Y* axis, and 12 mg for the *Z* axis. The average total drift is between 2 mg and 6 mg for all the DUTs and axes.

### 4.4. System Noise

Even after the filtering process, there is a significant level of noise in all DUTs. This noise makes it difficult to analyze the DUTs’ behavior in short times or with little temperature variations. All the data transmissions are digital; therefore, the noise is generated in the accelerometers internal electronics. This noise could be affected by the accelerometer configuration; however, the slowest sample rate—which is the one in use—should be the least noisy. This noise can only be reduced with more restrictive software filters. To analyze the noise levels of the DUTs, the tenth hour of the cool start test is chosen, since it has low drifts (see Table 4).

In all six DUTs, the *Z* axis has a higher noise than the other two axe, typically 1.8 mg against 1.6 mg on the *X* axis and 1.3 mg on the *Y* axis. All the DUTs have a maximum noise between 0.95 mg and 2.05 mg.

## 5. Analysis

Theoretically the temperature dependent phenomena (Section 4.1 and Section 6.2) can be compensated, or at least reduced, by knowing the characteristic parameters TDB and TDSF from Equation (4). To obtain these parameters the most common solution is to use all the available data and fit a polynomial equation with the least squares method. This can be performed by computers with specialized software, but not by low-cost MCUs or CPUs. All sensors and axes must be calibrated individually; therefore, an easier algorithm should be used. By selecting only the relevant data according to the temperature profile, the computing cost can be drastically reduced.

The reference temperature, $T_{R}$, is the one in which the sensor is considered to have no drift and the value read, *X*, is the actual value $X_{0}$ (see Equation (8)). According to the manufacturer, the sensors are calibrated at 25 °C, so this is taken as the reference temperature.

(8) $$X\left(T_{R}\right)=X_{0}+\left(T_{R}-T_{R}\right)\left(TDB+TDSF\cdot X_{0}\right)\Rightarrow X\left(T_{R}\right)=X_{0}.$$

When the test is performed in a stable position, it is correct to assume that $X_{0}$ in each axis is constant. Therefore, the sensitivity component of the thermal drift ($TDSF\cdot X_{0}$) is also constant. This means that, for each static position, the thermal drift of the sensor is directly proportional to the temperature. A unique thermal drift parameter, $TD_{X_{0}}$, can be obtained for any specific orientation tested (see Equation (9)).

(9) $$X_{0}=const\Rightarrow TDSF\cdot X_{0}+TDB=const=TD_{X_{0}}.$$

And:

(10) $$X=X_{0}+\left(T-T_{R}\right)\cdot TD_{X_{0}}\Rightarrow TD_{X_{0}}=\frac{X-X_{0}}{T-T_{R}}\Rightarrow TD_{X_{0}}=\frac{\Delta X}{\Delta T}.$$

The thermal drift parameter specific to one orientation, $TD_{X_{0}}$, can be obtained as the ratio between the data variation and the temperature variation 10. These variations are always proportional, since $X_{0}$ does not change, and allow any combination of two accelerations and their two respective temperatures to be used to compute $TD_{X_{0}}$.

(11) $$TD_{X_{0}}=\frac{X_{1}-X_{2}}{T_{1}-T_{2}}.$$

**Figure 10:**
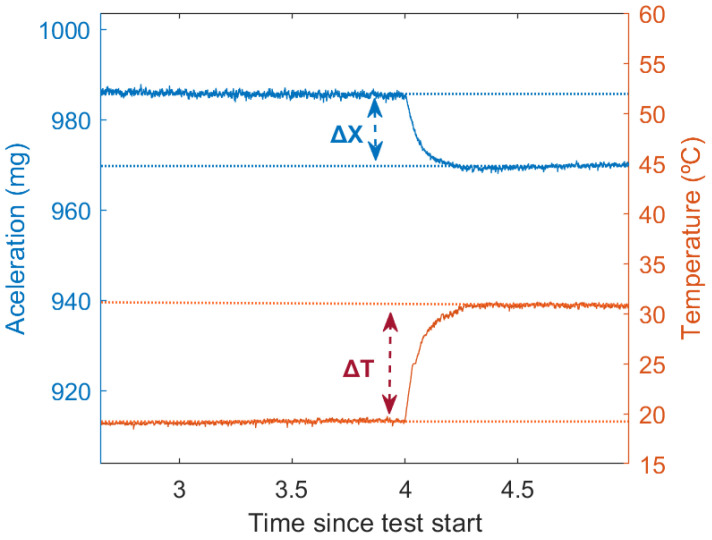
Values extracted for the $TD_{X_{0}}$ computing for one step.

For each temperature step of each test, one $TD_{X_{0}}$ is computed using Equation (11) (see Figure 10), this will reduce the computing load compared to using all the data points. Each temperature step will result in one value of $TD_{X_{0}}$. Finally, all the obtained values for the same axis and test are averaged.

If the temperature variations between the steps were too low, the acceleration variations would also be low, and Equation (11) would approximate to 0/0. The noise of both signals would lead to incorrect data that does not represent the sensors behavior. In our case, all the temperature variations exceed 10 °C and are considered reliable.

### Temperature Drift of Bias and Temperature Drift of Scale Factor

**Figure 11:**
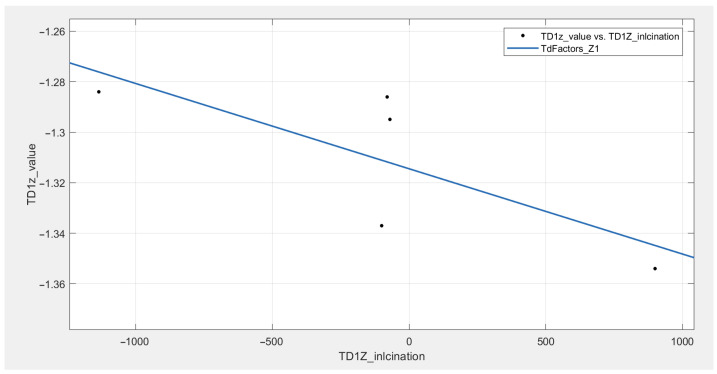
TDB and TDSF computing from the $TD_{X_{0}}$ values (DUT #1, *Z* axis).

Once the six $TD_{X_{0}}$ for each axis and DUT are known, they can be used to compute TDB and TDSF. The points can be represented as a straight line, where TDB is the bias, and TDSF is the slope (see Figure 11). Both parameters can be obtained with a linear regression. It is also possible to compute the TDB as the average of the $TD_{X_{0}}$ when $X_{0}$ is approximately 0 mg and TDSF as the slope between the $TD_{X_{0}}$ at ±1000 mg. This could lead to even lower computational costs.

The TDB sign and values appears to be random, with values up to 1.3 mg/°C. DUT #1 has the largest temperature drifts, with 1.3 mg/°C in two axes. DUTs #2 to #5 show lower temperature drifts, with typical values around 0.4 mg/°C. The control DUT also shows large temperature drifts, up to 1 mg/°C. All computed TDSF are negative in sign but random in value. The *Z* axes do not show greater temperature drifts compared to the *X* axes. The *Y* axis has lower drifts than the others. The parameters obtained for each axis are shown in Table 5.

## 6. Compensation

Once the accelerometers thermal behavior is characterized, it is possible to compensate for thermal drift. The thermal behavior formula (Equation (4)) can be rearranged to obtain the real acceleration from the raw acceleration and the temperature (see Equation (12)).

(12) $$X_{0}=\frac{X-\Delta T\cdot TDB}{1+\Delta T\cdot TDSF}.$$

**Figure 12:**
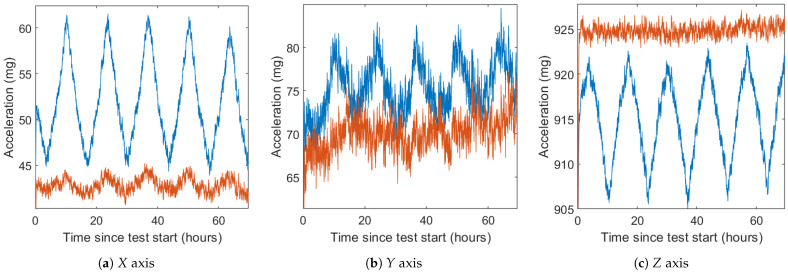
Filtered accelerations of DUT #1 before (in blue) and after (in red) compensation in the verification test.

Data compensation adds noise to the signals by combining acceleration and temperature noise. An exponential filter with α=0.001—response time on the order of 10 min—is applied to reduce all the noise and better appreciate the effect of compensation, as shown in Figure 12. This can be done in tilt measurement applications, where response time is not a concern.

### 6.1. Thermal Drift

To show the effect of compensation, the verification test will be analyzed before and after compensation. Thermal drift causes variations in the acceleration value; therefore, the compensated data should show lower variations in its value. To quantify the improvement due to compensation, the standard deviation and the maximum error will be used as indicators. These parameters show the data immunity to temperature variations and, therefore, the performance of the compensation technique. The verification test (explained in Section 3.3) is chosen as it has slower temperature variations than the calibration tests, closer to those expected in application. In addition, it has not been used to compute the TDB and TDSF values. The maximum temperature difference during this test was 20 °C; therefore, all the subsequent results are conditioned by this parameter.

The standard deviation, σ, is a measure of the variation or dispersion of a data set. A smaller standard deviation indicates that the values tend to be close to the mean. In our case, it implies that temperature variations do not affect the acceleration value, that is, thermal drift is reduced. Standard deviation for all DUTs is shown in Table 6.

The *X* axes have, when uncompensated, the biggest σ of all three. They have an average value of 2.90 mg and two of the DUTs (including the control DUT) are over 4 mg. After compensating, they become much more stable, with an average σ of 0.80 mg, 72% lower. DUT #2 slightly deteriorates its performance, although it stays at 1.04 mg.

The *Y* axes show less improvement than the *X* axes, σ goes from 1.87 mg to 1.34 mg. This represents a 28% improvement. The lower improvement can be related to the already lower σ when uncompensated. None of the DUTs deteriorated their behavior after compensation.

The uncompensated *Z* axes show a similar drift to the *X* axes, with an average σ of 2.80 mg. After compensation, it decreases to an average of 1.09 mg, 61% lower. All DUTs, except #2, had an uncompensated σ over 2 mg; no compensated data exceeds that value. Overall, no compensated axis exceeds 2 mg of deviation, and half of them are below 1 mg of σ.

All improvement percentages are computed as the difference between compensated and uncompensated, and divided by the uncompensated value.

The maximum deviation can be obtained as the difference between the higher and the lower values and is related to the maximum expected error. These data are shown in Table 7.

In this case, the improvement is slightly lower than that of the standard deviation This can be related to the fact that the maximum error only takes into account the two worst values, while the standard deviation takes into account all the data.

Similar to the standard deviation, the *X* axis shows the largest average improvement, 61%. The *Z* axis also shows a great improvement, greater than 50%. The *Y* axis shows an average improvement of 20%. In all three cases, the average uncompensated maximum deviation was over 10 mg. Compensation reduces this value in the *X* and *Z* axes to below 5 mg, and to 8.3 mg in the *Y* axis.

The best improvement is on the *X* axis of the control DUT, with an 81% improvement. The only axis that does not reduce its maximum error is the *X* of DUT #2.

### 6.2. Self-Heating Drift

**Figure 13:**
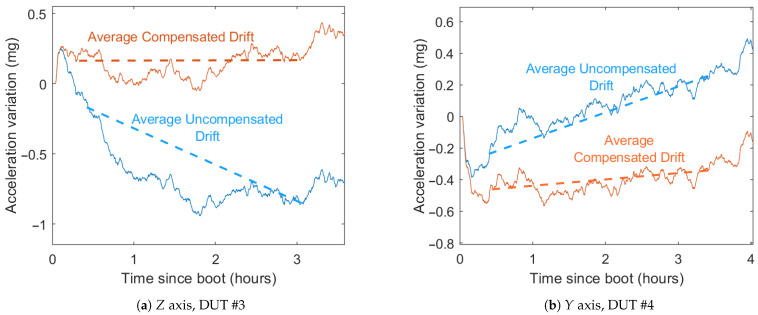
Cool start drifts before and after compensation.

In some cases, the initial drift caused by the self-heating can also be reduced with this method (see Figure 13). This self-heating drift is most dominant in the first three hours after a cool start, as shown in Section 6.2. The drift during the first 30 min after start is still high in many units and it is not advisable to rely on these measurements.

To determine the compensation performance in the self-heating effect, the average drift between 30 min and three h after the test start is measured in Table 8.

All three axes of the six DUTs reduce their self-heating drift, except for the *X* axis of the second DUT. That deteriorated drift gets increased to 0.112 mg/h, which is still a low value compared to the others. This deterioration is due to the fact that, in this DUT, thermal drift and temporal drift act in opposite directions, compensating for each other. When self-heating drift is compensated for, the temporal drift remains, and the end result is a drift greater than the original.

The average drift in the *X* axes is reduced from 0.62 mg/h to 0.281 mg/h, a 54% improvement. In *Y*, it gets reduced from 0.689 mg/h to 0.512 mg/h, a 25% improvement. And, in the *Z* axes, it goes from 0.828 mg/h to 0.426 mg/h, a 48% improvement.

### 6.3. Improvement as Inclinometer

**Figure 14:**
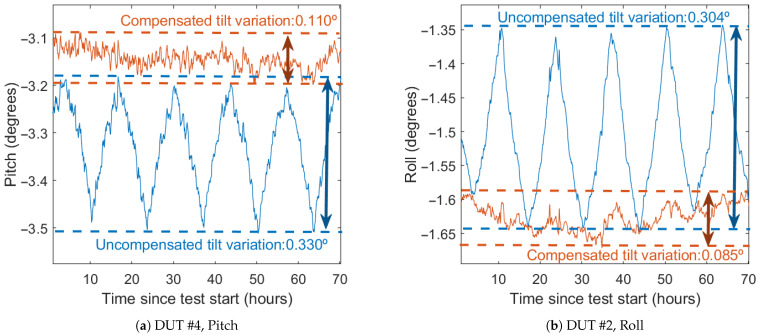
Euler angles before and after calibration.

To measure the performance of the compensation in a tilt measurement application, the Euler angles—pitch (Equation (2)) and roll (Equation (3))—are computed from both the uncompensated and compensated data (see Figure 14). Before computing the Euler angles, the six DUTs have been calibrated with a six-position static test [28] using a surface plate and an orthogonal cube. This is used to compensate for bias, sensitivities, and misalignments of each axis (Equation (1)), allowing for more reliable tilt measurements.

Figure 14 and Table 9 and Table 10 show the result of the calculation of the Euler angles before and after the thermal compensation during the verification test. As with acceleration, both standard deviation and maximum error are used as compensation performance indicators.

The average standard deviation without compensation is 0.151° for pitch and 0.092° for roll. The worst results before compensation are in the DUT #1 and the control DUT, both in the pitch angle. After compensation, this deviation is reduced to 0.040° and 0.036° respectively (85% and 88% improvement). In general, the average standard deviation with compensation is 0.036° for pitch (76% lower than uncompensated) and 0.059° for roll (36% lower). The compensated deviation is never greater than 0.06° for pitch and 0.1° for roll, bringing the results of the all DUTs closer together. Most of the DUTs with already low standard deviation (lower than 0.1°) do not show significant improvement, with two DUTs showing a slight deterioration, although they stay lower than 0.1°.

The maximum detected deviation exhibits a similar behavior to the standard deviation, with a maximum deviation detected in the pitch of the DUT #1 and the control DUT. Average maximum deviation decreases from 0.592° to 0.178° in pitch (70% lower) and from 0.433° to 0.319° in roll (26.3% lower). The maximum error after compensation is never greater than 0.3° for pitch and 0.6° for roll.

## 7. Discussion

### 7.1. Methods Comparison

The proposed method is considered similar to the surface calibration proposed by Ruzza et al. The main difference between them methods is that, in that case, a generic polynomial equation is used and the calibration parameters are adjusted without taking into account their physical meaning. In our case, a formula derived from the theoretical behavior of the sensor is used. This allows fewer compensation parameters to be used, reducing the amount of information needed to obtain them.

The advantage of using a generic polynomial fit is that it has a greater ability to compensate for behaviors that have not been previously taken into account, as long as they are not of a higher order than the polynomial equation.

The great advantage of the proposed method is the less amount of data necessary to obtain the calibration parameters and a clear relationship of them with a physical characteristic of the sensor. A lower computational cost also facilitates the recalibration of systems even in application.

Since both methods are similar, the parameters of one can be obtained with respect to the other by means of a regression. In Appendix A, the TDB and TDSF corresponding to the calibration surfaces used in Reference [23] have been calculated. The maximum relative error that is committed in each case when making the adjustment has also been calculated.

The surface parameters that cannot be adjusted with the proposed method are those of the second order of both the TDB and TDSF ($p_{20}$ and $p_{02}$). This is the main cause of the residuals when trying to match one method with the other. It can be seen that the greater these parameters are, the greater the maximum error and RMSE are. When these parameters are small, the residuals are reduced, allowing a good fit between both methods. If the behavior of the TDB and TDSF is of the first order, the efficiency of both methods is similar.

### 7.2. Application of the Thermal Calibration Algorithm

As with most calibration methods, using more data produces more reliable results, as noise and other random errors are averaged. It is recommended to use at least six calibration orientations and the full working range of the sensors for best results. It is considered that a viable option would be to perform the thermal calibration simultaneously with the mechanical calibration of the axes (bias, sensitivity, and cross-axis sensitivity).

In theory, with just two samples of $TD_{X_{0}}$ (in different orientations), the two calibration parameters (TDB and TDSF) can be obtained. Furthermore, only two temperatures are required in each orientation to obtain the corresponding $TD_{X_{0}}$. Therefore, the fastest theoretical method to obtain the complete thermal calibration of the accelerometer requires only two orientations and two temperature variations (see Appendix B). The effectiveness of this method has not been tested. Any noise or temporary effects could affect the calibration, so it is recommended to use a greater number of points.

The performance of thermal compensation with this method is considered adequate. Considerable improvements are achieved in cases where the thermal drift is higher. This allows the operation of all units after calibration to be more uniform. This is because the greater the thermal drift, the easier it is to isolate and characterize it. If the thermal drift is very small, it mixes with the noise and cannot be compensated for. Therefore, effectively reducing noise in the signal could improve the efficiency of compensation, at the cost of increasing its complexity.

### 7.3. Typical Drifts

The TDB and TDSF data obtained in this work agree with the information provided by the manufacturer and other works. According to the manufacturer, the typical value of TDB is ±0.5 mg/°C, while the mean of the TDBs obtained in this work is 0.62 mg/°C. The technical information does not provide any information on the expected range of TDB, which in our case is ±1.3 mg/°C. The accelerometer tested by Ruzza et al. shows higher values, with a mean value of −1.24 mg/°C and a maximum of −1.67 mg/°C (values obtained by adjusting the methods in Appendix A).

In the case of TDSF, the mean values obtained are also close to the change in sensitivity indicated by the manufacturer (100 ppm/°C). In our case, the average value obtained is −160 ppm/°C with a maximum value of −398 ppm/°C. In the case of Ruzza et al., the average value obtained is −113 ppm/°C with a maximum of −191 ppm/°C. All the values obtained are negative, as indicated by theoretical studies. This means that the sensitivity always decreases when the temperature increases.

Furthermore, it can be observed that the drifts in the *X* axis are greater than in the *Y* axis both in this work and in that of Ruzza et al. [12]. This difference is quite remarkable in both studies, although the design of the two sensitive axes should be similar.

The control DUT has shown a similar level of thermal drift as the other units. This suggests that most of the thermal drift is due to internal factors (changes in stiffness) rather than factors related to the soldering process and possible surface tensions of the PCB.

### 7.4. Self-Heating

The initial drift has been reduced between 25% and 48%. Part of this drift is due to self-heating and part is due to the temporal drift, which cannot be eliminated by this method.

This improvement can be especially relevant for IoT devices to minimize the energy consumption. The self-heating drift compensation can lead to more reliable results after the systems wake up. If the MCU requires less time to achieve the same results, the energy consumption will be reduced accordingly.

### 7.5. Computing Time

The performance of the implementation of the proposed method can be compared with other techniques that also rely on polynomial equations. The computing time of three compensation algorithms in two different MCU are shown in Table 11.

In these low-cost MCUs, the time required for each method is on the order of tens of microseconds. The proposed method and the third order curve take a similar time to compute: 65.6 μs (Proposed Method) against 63.7 μs (Third Order Curve) in the ATmega328P and 27.7 μ against 34.1 μs in the SAMD21G18A. The second order surface takes approximately 78% more time to compute compared to the proposed method. This could be caused by the higher number of operations that have to be carried out.

In general, the proposed equation requires less computation time than high-degree polynomials, making it more suitable for low cost, low power, or time critical applications.

## 8. Conclusions

The thermal characteristic parameters, Temperature Drift of Bias and Temperature Drift of Scale Factor, are random in value and TDB also in sign, and can greatly affect the MEMS measurements when the temperature changes. Therefore, individualized calibration is essential for applications with thermal variations. This behavior is shown even before the soldering processes. According to the manufacturer, this process can also affect the calibration; therefore, the individual calibration must be carried out after the soldering process.

The proposed calibration method—relying on individual temperature drift coefficients for specific orientations—has been effective, particularly in the axes with the greater thermal drifts. This method has low computational and memory requirements, requiring small amounts of data and generating only two compensation parameters for each axis. To perform this calibration method only temperature variations, mechanical stability and one temperature sensor, usually integrated with the inertial sensor, are needed. This can lead to in-application calibration or recalibration.

The implementation of the calibration in a microcontroller unit is lightweight both in memory and computing cost. It has also shown that there is little difference compared to using a second-order surface, computationally more complex. The performance difference between the two resides mainly in the second order of the TDB.

In this paper, six tests were performed with 40 °C variations over six DUTs. With this data, the accelerometers were characterized. Another test was performed with temperature ramps of 20 °C to try out the effects of the compensation. After compensating the DUTs and computing the Euler angles, the average accuracy improvement was between 27% and 76%, depending on the angle and the method. In the DUTs with higher drifts, DUT #1 and CU, the improvements where up to 88% in thermal stability.

This technique allows the use of low cost MEMS accelerometers as inclinometers with a standard deviation lower than 0.1° (with 20 °C thermal variations). The effect of the self-heating drift is also compensated to some extent in the 20 min to 3 h segment after cool start.

## Figures and Tables

**Table 1:** Typical characteristics of the LIS3DSHTR MEMS accelerometer in the ±2 g range configuration (FS bit set to 000).

Parameter	Typical Value
Resolution	16 bits
Sensitivity	0.06 mg
Output data rate	3.125 Hz to 1.6 kHz
Sensitivity change vs temperature (TDSF)	0.01%/°C
Typical zero-g level offset accuracy	±40 mg
Zero-g level change vs. temperature (TDB)	±0.5 mg/°C
Acceleration noise density (Data Rate = 100 Hz)	150 μg/$\sqrt{\mathrm{Hz}}$

**Table 2:** Configuration of the studied units.

Register	Value (HEX)	Details
CTRL_REG4 (20 h)	17	ODR: 3.125 Hz. All axes active
CTRL_REG5 (24 h)	C0	Antialiasing: 50 Hz. FS: ±2 g. No self-test. 4-wire SPI

**Table 3:** Temporal drifts magnitude for each axis (mg/h).

	*X*			*Y*			*Z*		
Time	Avg.	Worst	C.U.	Avg.	Worst	C.U.	Avg.	Worst	C.U.
0–1 h	1.24	3.43	2.31	1.68	2.21	4.79	1.18	2.75	3.58
1–3 h	0.39	1.15	0.35	0.42	0.89	0.83	0.46	1.13	0.63
3–5 h	0.20	0.55	0.06	0.23	0.48	0.12	0.28	0.81	0.12
5–10 h	0.24	0.34	0.01	0.16	0.33	0.10	0.40	0.58	0.24
10–20 h	0.10	0.25	0.07	0.13	0.21	0.02	0.16	0.33	0.10

**Table 4:** Noise for each DUT and axis during the 10th hour of the cool start test (mg).

	*X*		*Y*		*Z*	
DUT	Standard	Max	Standard	Max	Standard	Max
	Deviation	Deviation	Deviation	Deviation	Deviation	Deviation
1	0.221	1.704	0.201	1.305	0.297	2.031
2	0.227	1.619	0.196	1.357	0.245	1.802
3	0.193	1.333	0.170	1.245	0.242	1.736
4	0.232	1.699	0.187	1.292	0.247	1.859
5	0.176	1.240	0.160	0.980	0.267	1.592
Control	0.224	1.678	0.216	1.445	0.261	1.699

**Table 5:** Computed temperature drifts parameters for each axis and DUT.

	*TDB* (mg/°C)			*TDSF* (ppm/°C)		
DUT	X	Y	Z	X	Y	Z
1	1.26	0.76	−1.32	−118	−44	−34
2	0.09	0.31	0.12	−42	−103	−188
3	0.3	−0.22	−0.3	−107	−188	−104
4	−0.44	0.19	−0.61	−128	−277	−60
5	0.39	−0.08	−0.72	−398	−65	−169
Control	−1.18	−0.21	−0.81	−134	−46	−226

**Table 6:** Standard deviation of the samples during the verification test (mg).

	*X*			*Y*			*Z*		
DUT	Uncomp.	Comp.	Impr.	Uncomp.	Comp.	Impr.	Uncomp.	Comp.	Impr.
1	4.66	0.77	83.48%	2.99	1.91	36.12%	4.49	0.63	85.97%
2	0.92	1.04	−13.04%	1.56	0.34	78.21%	0.72	0.77	−6.94%
3	2.49	1.26	49.40%	2.08	1.51	27.40%	2.52	1.28	49.21%
4	1.54	0.44	71.43%	1.78	1.61	9.55%	2.38	1.08	54.62%
5	2.28	0.63	73.68%	0.84	0.77	8.33%	3.47	1.95	39.77%
Control	5.51	0.69	87.48%	1.96	1.88	4.08%	3.21	0.68	78.82%
Avg.	2.90	0.80	72.41%	1.87	1.34	28.46%	2.80	1.09	61.11%

**Table 7:** Maximum deviation of the DUTs during the verification test (mg).

	*X*			*Y*			*Z*		
DUT	Uncomp.	Comp.	Impr.	Uncomp.	Comp.	Impr.	Uncomp.	Comp.	Impr.
1	17.63	4.63	73.74%	18.51	13.81	25.39%	18.44	4.26	76.90%
2	5.41	6.31	−16.64%	6.08	1.92	68.42%	4.07	3.78	7.13%
3	11.07	6.26	43.45%	11.28	8.41	25.44%	11.21	6.25	44.25%
4	7.12	3.41	52.11%	11.10	10.66	3.96%	10.19	4.77	53.19%
5	9.44	3.48	63.14%	4.96	4.68	5.65%	13.27	7.47	43.71%
Control	23.74	4.45	81.26%	10.79	10.39	3.71%	13.06	3.47	73.43%
Avg.	12.40	4.76	61.64%	10.45	8.31	20.49%	11.71	5.00	57.29%

**Table 8:** Average acceleration drift between 30 min and 3 h after cool start (mg/h).

	*X*			*Y*			*Z*		
DUT	Uncomp.	Comp.	Impr.	Uncomp.	Comp.	Impr.	Uncomp.	Comp.	Impr.
1	1.636	0.896	45.2%	1.220	0.760	37.7%	−1.572	−0.804	48.9%
2	−0.088	−0.112	−27.3%	0.344	0.176	48.8%	−0.044	−0.008	81.8%
3	0.280	0.100	64.3%	−0.628	−0.520	17.2%	−0.244	0.020	91.8%
4	−0.364	−0.108	70.3%	0.136	0.024	82.4%	−1.344	−0.956	28.9%
5	0.652	0.420	35.6%	−0.536	−0.476	11.2%	−0.724	−0.132	81.8%
Control	−0.716	0.052	92.7%	−1.268	−1.116	12.0%	−1.040	−0.636	38.8%
Avg.	0.623	0.281	54.8%	0.689	0.512	25.7%	0.828	0.426	48.6%

**Table 9:** Euler angles standard deviation before and after compensation (degrees).

	Pitch			Roll		
DUT	Uncomp.	Comp.	Impr.	Uncomp.	Comp.	Impr.
1	0.268	0.040	85.0%	0.164	0.091	42.2%
2	0.026	0.036	−36.9%	0.084	0.016	80.0%
3	0.119	0.058	50.7%	0.111	0.071	35.6%
4	0.085	0.019	77.6%	0.081	0.067	17.1%
5	0.104	0.027	73.7%	0.035	0.023	33.3%
Control	0.302	0.036	88.1%	0.076	0.085	−11.8%

**Table 10:** Euler angles max error deviation before and after compensation (degrees).

	Pitch			Roll		
DUT	Uncomp.	Comp.	Impr.	Uncomp.	Comp.	Impr.
1	0.953	0.194	79.5%	0.824	0.529	35.7%
2	0.142	0.173	−21.9%	0.304	0.085	72.0%
3	0.507	0.274	45.9%	0.539	0.356	33.9%
4	0.330	0.110	66.6%	0.393	0.351	10.6%
5	0.405	0.140	65.4%	0.158	0.129	18.1%
Control	1.213	0.177	85.4%	0.381	0.465	−22.0%

**Table 11:** Computing time for the compensation methods in different MCUs.

MCU	Proposed Method	Second Order Surface	Third Order Curve
ATmega328P	65.6 μs	117.6 μs	63.7 μs
ATSAMD21G18A	27.7 μs	49.1 μs	34.1 μs

## Abbreviations

The following abbreviations are used in this manuscript:

MEMS	MicroElectroMechanical System
TDB	Temperature Drift of Bias
TDSF	Temperature Drift of Scale Factor
MCU	Microcontroller Unit
DUT	Device Under Test

## Appendix A

The second-order surfaces have been generated throughout the full working range, both in accelerations (±1 g) and in temperatures (−10 °C to +70 °C).

**Table A1:** Fit using the proposed formula of second-order calibration surfaces.

	Second Order Surface		Proposed Method		Goodness of Fit		Relative Maximum
	$p_{00}$, $p_{10}$, $p_{11}$	$p_{01}$, $p_{20}$, $p_{02}$	*TDB*	*TDSF*	$R^{2}$	RMSE	Error
	571.5	$1.56\times 10^{-3}$					
Case 1	−20.23	−0.104	−1.64	−70.7	0.9848	4.904	0.575%
	$-6.93\times 10^{-5}$	$6.32\times 10^{-9}$					
	582.9	$7.23\times 10^{-4}$					
Case 2	−21.29	$-7.898\times 10^{-2}$	−1.61	−70.5	0.9893	4.045	0.557%
	$-6.362\times 10^{-5}$	$-7.354\times 10^{-8}$					
	354	$5.747\times 10^{-3}$					
Case 3	−13.96	$-2.931\times 10^{-3}$	−0.86	−183.8	0.9990	0.663	0.135%
	$-1.915\times 10^{-4}$	$-5.363\times 10^{-8}$					
	338.1	$4.439\times 10^{-3}$					
Case 4	−12.82	$-3.299\times 10^{-2}$	−0.91	−154.4	0.9936	1.789	0.237%
	$-1.579\times 10^{-4}$	$-1.455\times 10^{-8}$					

## Appendix A

Theoretically faster calibration method:

1. Place the accelerometer with a pitch and roll of approximately 35°. In this way, the accelerations in the 3 axes are about 577 mg, achieving the maximum study range for the three axes.
2. Generate a temperature of 25 °C and record the acceleration measurements. This is the $X_{0_{1}}$ for each axis.
3. Generate a thermal variation, for example, 20 °C.
4. Use these new accelerations to compute the $TD_{1}$ with Equation (11) for each axis.
5. Without lowering the temperature, invert the accelerometer: pitch and roll angles at approximately −35°.
6. Record acceleration.
7. Generate again the temperature of 25 °C, these accelerations are $X_{0_{2}}$.
8. Use these accelerations to obtain the $TD_{2}$.
9. TDSF is calculated as the difference of $TD_{1}$ and $TD_{2}$ divided between the difference of $X_{0_{1}}$ and $X_{0_{2}}$ (see Equation (A1)).
10. TDB is calculated as any TD minus the TDSF multiplied by the corresponding $X_{0}$ (see Equation (A2)).
  (A1) $$TDSF=\frac{TD_{1}-TD_{2}}{X_{0_{1}}-X_{0_{2}}},$$
  (A2) $$TDB=TD_{1}-TDSF\cdot X_{0_{1}}=TD_{2}-TDSF\cdot X_{0_{2}}.$$

In this case, it is recommended to use Euler angles of 35° to maximize the accelerations range in all three axes with only two orientations, although it is possible to perform this technique with any two orientations. It is also important to use only the accelerometers measurements for the calculation of TDB and TDSF, since misalignments between the sensitive axes and the accelerometer body could cause deviations in the parameters obtained if external references are used.
